# Insemination with border disease virus-infected semen results in seroconversion in cows but not persistent infection in fetuses

**DOI:** 10.1186/s12917-018-1472-6

**Published:** 2018-05-16

**Authors:** Ueli Braun, Fredi Janett, Sarah Züblin, Michèle von Büren, Monika Hilbe, Reto Zanoni, Matthias Schweizer

**Affiliations:** 1 Department of Farm Animals, Vetsuisse Faculty, University of Zurich, Zurich, Switzerland; 2 Institute of Veterinary Pathology, Vetsuisse Faculty, University of Zurich, Zurich, Switzerland; 3 Institute for Virology and Immunology, and Department of Diseases and Pathobiology, Vetsuisse Faculty, University of Bern, Bern, Switzerland

**Keywords:** Border disease virus, BDV, Semen, Insemination, Seroconversion, Pestivirus, Cattle, Persistent infection

## Abstract

**Background:**

This study examined various health variables in cows after artificial insemination with Border disease virus (BDV)-infected semen and the occurrence of persistent infection in ensuing fetuses. Five cows were inseminated (day 0) with BDV-infected semen as well as with semen from a fertile Eringer bull. One cow, inseminated with virus-free semen only, served as a control. Clinical examination, assessment of eating and rumination activities, measurement of intraruminal temperature and leukocyte count were used to monitor the health of the cows. Blood samples were collected at regular intervals for the detection of viral RNA and antibodies against BDV, and the cows were slaughtered on day 56. The uteri, placentae and fetuses were examined macroscopically, histologically, immunohistochemically and by means of molecular methods for the presence of pestiviruses.

**Results:**

The demeanour, eating and rumination activities and intraruminal temperature were not affected by insemination with BDV-infected semen, whereas the total leukocyte and lymphocyte counts dropped transiently and were significantly lower on day 6 than on day 0. Seroconversion occurred by day 28 in the five infected cows but not in the control cow. The uteri, placentae and fetuses had no macroscopic or histological lesions, and immunohistochemical examination and RT-PCR were negative for pestiviruses.

**Conclusions:**

The findings showed that cows inseminated with BDV-infected semen seroconverted and fetuses thus produced were not persistently infected. Transmission of BDV to cattle through infected semen, therefore, seems to be of minor importance.

## Background

It is well known that Border disease virus (BDV) can be transmitted from sheep to cattle under natural conditions as well as experimentally [1–9]. Transmission of BDV from a persistently-infected calf to a seronegative heifer in early pregnancy through direct contact was recently reported [10]. Seroconversion in cows was also observed after artificial insemination with semen from a bull persistently infected with BDV even though the cows failed to conceive because of poor semen quality [11]. Twice-daily monitoring showed no abnormalities in demeanour, appetite and rectal temperature of the cows [11] but temperature spikes of short duration may have been missed. Calves had spikes in intraruminal temperature as early as 2 days after intranasal infection with bovine viral diarrhoea (BVD) virus [12]. The mechanism by which BDV affects the leukogram in cattle is not known, but cattle with BVDV infection have leukopenia and lymphopenia [13]. The goal of the study was to determine whether artifical insemination of cows with BDV-infected semen results in persistently infected offspring. The effect of using BDV-infected semen on the health of recipient cows was also investigated by evaluation of general well-being and by determining whether viraemia, pyrexia and/or seroconversion occured.

## Methods

### General procedure

Cows were artificially inseminated using semen from a young bull persistently infected with BDV. Eating and rumination variables were used as proxy for the health status and monitored using a pressure sensor that was incorporated into a halter [14, 15], temperature was measured using an intraruminal temperature-recording bolus, and blood samples were collected to determine the leukocyte count and the presence of BDV virus and antibodies. The cows were slaughtered 56 days after insemination, and the uteri, placentae and fetuses underwent macroscopic and histological examination and molecular testing for BDV.

### Animals

Six healthy non-pregnant Swiss Braunvieh cows, 2.8 to 6.2 years (3.8 ± 1.1 years) of age, were used. The cows originated from several farms and had been sold for slaughter because of unsufficient milk production. In all cows, skin biopsy samples tested negative for pestivirus antigen and blood samples were negative for pestivirus antibody. Five cows (nos. 1, 3, 4, 5, 6) served as experimental cows and one (no. 2) served as a control.

### Acclimation, oestrus synchronisation, artificial insemination and duration of infection phase

The cows were kept in quarantine during the entire study period and were acclimatised for 10 days. All cows underwent oestrus synchronisation and were artificially inseminated twice 24 h apart (day 0 and day 1 of the infection phase) using semen from a young bull persistently infected with BDV [10, 11]. The genome of the virus causing persistent infection (Sub-Genotype BDSwiss) has since been sequenced [16]. The semen had a high virus titre at 2.51 × 10^8^ TCID_50_ (50% tissue culture infective dose)/ml and 1.44 × 10^6^ TCID_50_/10^6^ sperm cells. Because cows inseminated with infected semen failed to become pregnant in an earlier study [11], the cows of the present study were concurrently inseminated with virus-free semen from a bull of the Eringer breed with proven fertility. The goal was to transmit BDV with the semen from the persistently-infected bull and to impregnate the cows with the semen from the fertile bull. The control cow was inseminated with semen from the Eringer bull. The infection phase ended on day 56 when the cows were slaughtered.

### Clinical examination and monitoring of eating, rumination and intraruminal temperature

All cows underwent twice-daily clinical examination, which included determination of general demeanour, appetite, rectal temperature, heart rate, respiratory rate, consistency of faeces and the presence of ocular and mucous membrane abnormalities as well as ocular and nasal discharge. To detect possible viraemia-associated reduction in eating and rumination, the cows were fitted with a custom-made halter equipped with a pressure sensor in the noseband (MSR Electronics GmbH, Seuzach, Switzerland) to record jaw movements during eating and rumination [14, 15]. Eating and rumination times, number of regurgitated boluses per day and the number of chewing cycles per bolus were recorded from 5 days before until 15 days after insemination. Body temperature was recorded using an intraruminal temperature bolus (ThermoBolus® Large, San’Phone®, Medria Technologies, Châteaubourg, France). Measurements were made twice hourly and transmitted to a server. The system was programmed to send a text message when temperatures of ≥ 40.5 °C were recorded to initiate blood sampling for the detection of viral RNA.

### Haematological and virological examinations

Blood samples were collected every other day starting at day − 2 (2 days before the first insemination) until 24 days after insemination for haematological evaluation. Evacuated EDTA tubes (Vacuette, Greiner Bio-One GmbH, Kremsmünster, Oesterreich) were used for blood collection. A haemogram that included a total leukocyte count and leukocyte differential were carried out for each blood sample. The blood samples were examined for viral RNA by means of real-time RT-PCR. RNA from leukocytes was isolated using the QIAamp RNeasy blood mini kit (Qiagen AG, Hombrechtikon, Switzerland), and RT-PCR was done using the Qiagen QuantiTect Probe One Step RT-PCR Kit (Qiagen) according to the manufacturer’s instructions. The RT-PCR reactions were run on a thermocycler ABI 7300 (Applied Biosystems, Rotkreuz, Switzerland) with primers in the 5′-untranslated region (5’-UTR) which is used in our Swiss reference laboratory for ruminant pestiviruses and GAPDH as internal control [17]. The amount of viral RNA in the sample was expressed in Ct (cycle threshold) values; values of ≤ 30 were considered positive and values of > 30 up to < 45 as weakly positive. An in-house ELISA [18, 19] was used to detect pestivirus-specific antibodies in serum samples from the cows. This was done twice, 9 days apart, during the acclimation phase and eight times, 7 days apart, during the infection phase (days 7, 14, 21, 28, 35, 42, 49, 56), starting on day 0. Optical density (OD) values > 30% in relation to a standard serum were considered positive.

Blood samples collected on day 56 with a positive ELISA result underwent a cross-neutralisation test to identify the pestivirus species that induced the antibody response [20]. Briefly, sera were diluted tenfold in Eagles minimal essential medium and inactivated for 30 min at 56 °C. The sera were then further diluted in two-fold steps and incubated in 96-well plates for 1 h with a predetermined dose of BDV (BDSwiss, R9336/11, isolated from blood of the persistently-infected bull) or with BVD virus (BVDV-1a: R1935/72). Subsequently a suspension of embryonic bovine turbinate cells was added to each well and further incubated for 4 to 5 days, and the cells were then examined for pestivirus using immunoperoxidase staining. A difference in neutralisation titres for BVDV and BDV that was at least four-fold was considered significant, whereas a ratio of lower than 4 was considered as ‘indeterminate’ [20].

### Postmortem examination

All cows were slaughtered in the slaughterhouse of the Faculty on day 56, and the ovaries, uteri and placentae of the cows and skin, multiple bones, brain, heart, lungs, kidneys and intestinal organs of the fetuses were examined macroscopically and histologically. Monoclonal antibodies were used for immunohistochemical examination of fetal skin and organs (cryostat and paraffin sections) and uteri of the cows (paraffin sections) [21]. Cryostat sections were incubated with the BVD-specific antibody Ca3/34-C42 (dilution 1:100; Labor Dr. Bommeli AG, Bern, Switzerland) and the pestivirus-specific antibody C16/1/2 (Institute for Virology, University of Veterinary Medicine Hannover; kindly provided by Sophia Austermann-Busch, European Reference Laboratory for Classical Swine Fever and World Organisation for Animal Health). Paraffin sections were incubated with the pestivirus-specific antibody 15c5 (dilution 1:10,000, E. Dubovi, New York State College of Veterinary Medicine, Cornell University, USA) and the BVDV-specific antibody C42 (dilution 1:400, Prof. Moennig, Institute for Virology Hannover). Primary incubation was followed by incubation with the secondary antibody (DAKO Cytomation, EnVision+™, peroxidase, mouse, K 4001, Zug, Switzerland) and staining with AEC chromogens (DAKO, 3-Amino-9-Ethyl-Carbazole, K 3464). Positive and negative control slides were included in each run.

Samples of fetal thymus and small intestines, fetal and maternal placenta and uterine tissue of non-pregnant cows were examined for viral RNA. Tissue samples were frozen at − 80 °C, cut in small pieces with a scalpel and homogenised using the QIAshredder from the QIAamp RNeasy blood mini Kit (Qiagen) for isolation of total RNA. Virus detection was done by means of RT-PCR analogous to the method described for viral RNA detection in blood.

### Pedigree analysis

Fetal tissue samples were subjected to genotyping to determine the sire (Institute for Reproduction of Farm Animals, Schönow, Germany).

### Statistical analysis

The program IBM SPSS Statistics 24 (IBM Corporation) was used for analysis, and results were given as frequencies, means and standard deviations. A repeated measures ANOVA with Bonferroni correction and paired t-tests were used to analyse the profiles of eating and rumination variables, intraruminal temperature and white blood cell counts. Differences were considered significant at *P* < 0.05.

## Results

### Clinical findings

**Fig. 1:**
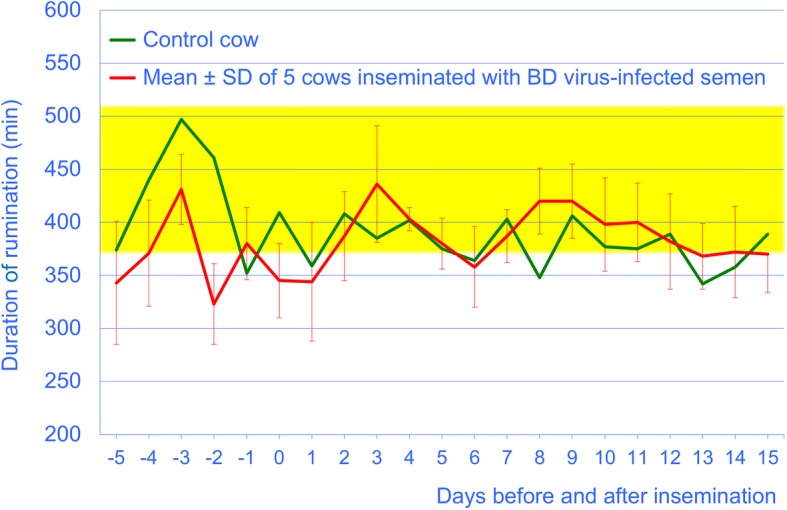
Daily rumination time from 5 days before until 15 days after insemination in 5 cows artificially inseminated with BDV-infected semen (mean ± SD) and 1 control cow. The yellow bar represents the reference interval (370 to 511 min) established in 300 healthy cows [15]

**Fig. 2:**
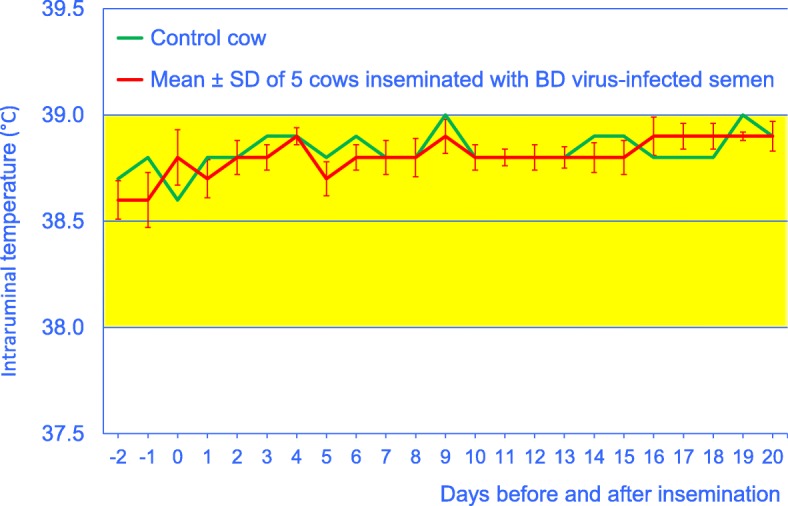
Intraruminal temperature from 2 days before until 20 days after insemination in 5 cows artificially inseminated with BDV-infected semen (mean ± SD) and 1 control cow. The yellow bar represents the reference interval (38.0 to 39.0 °C) for the rectal temperature [32]

Insemination with BDV-infected semen did not affect the general demeanour of the cows, the duration of eating and rumination (Fig. 1), the number of regurgitated boluses per day or the number of chewing cycles per bolus. The mean intraruminal temperature varied within the reference range and did not differ significantly before (38.6 ± 0.11) and after insemination (38.8 ± 0.09 °C, Fig. 2).

### Leukocytes and thrombocytes

**Fig. 3:**
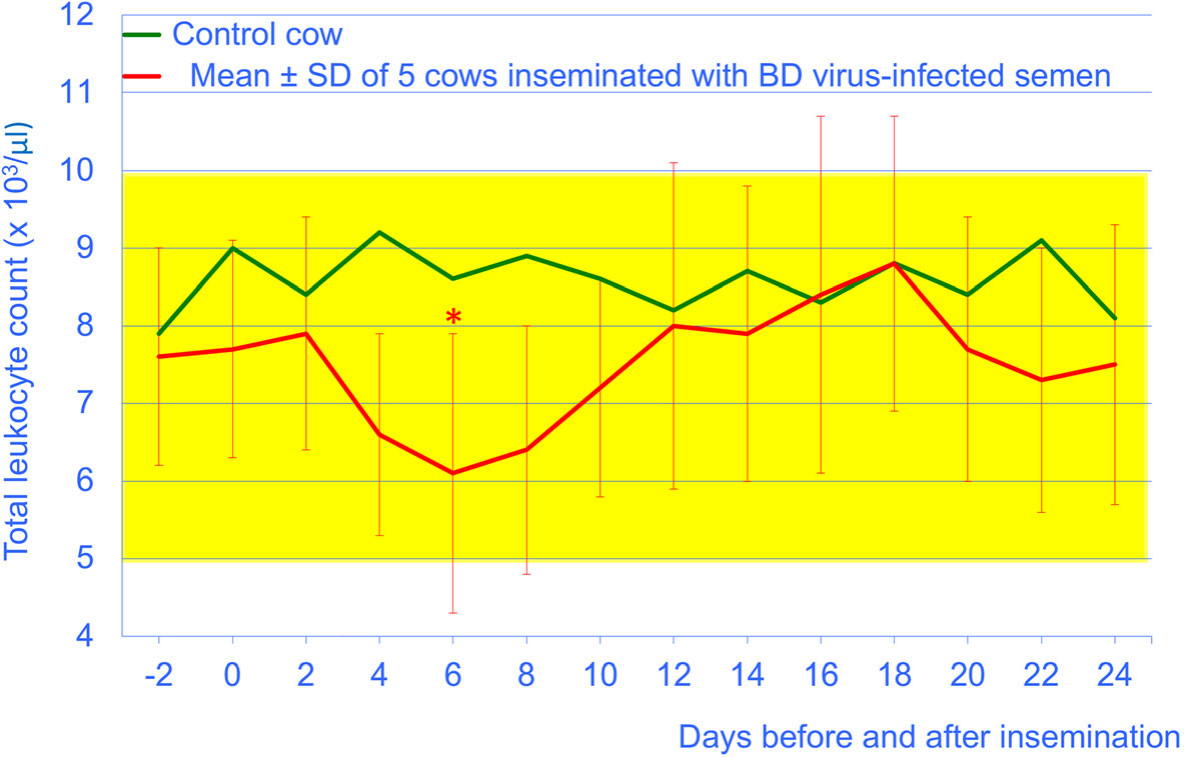
Total leukocyte count from 2 days before until 24 days after insemination in 5 cows artificially inseminated with BDV-infected semen (mean ± SD) and 1 control cow. The yellow bar represents the reference interval (5 to 10 × 10^3^/μl blood) [33], * = different from day 0 (*P* < 0.05)

**Fig. 4:**
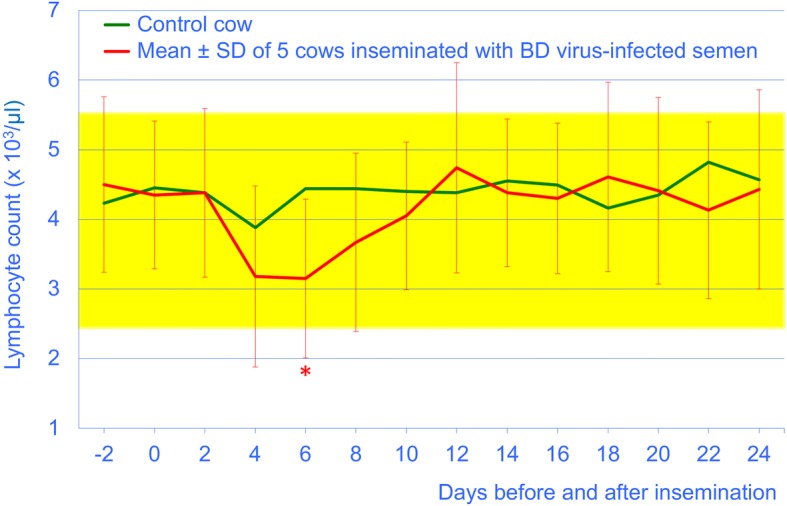
Lymphocyte count from 2 days before to 24 days after insemination in 5 cows artificially inseminated with BDV-infected semen (mean ± SD) and 1 control cow. The yellow bar represents the reference interval (2.5 to 5.5 × 10^3^/μl blood) [33], * = different from day 0 (*P* < 0.05)

The total leukocyte and lymphocyte counts of the experimental cows remained within the reference intervals throughout the study period. They decreased significantly from day 0 to day 6 (*P* < 0.05), after which time they returned to pre-insemination levels (Figs. 3 and 4). The thrombocyte counts did not differ before and after insemination and were in the reference interval in all cows (not shown).

### Virus detection in blood and seroconversion

With one exception, viral RNA was not detected in any blood samples before and in the first 24 days after insemination. Cow no. 6 had a weakly positive Ct value of 41.5 on day 24.

**Fig. 5:**
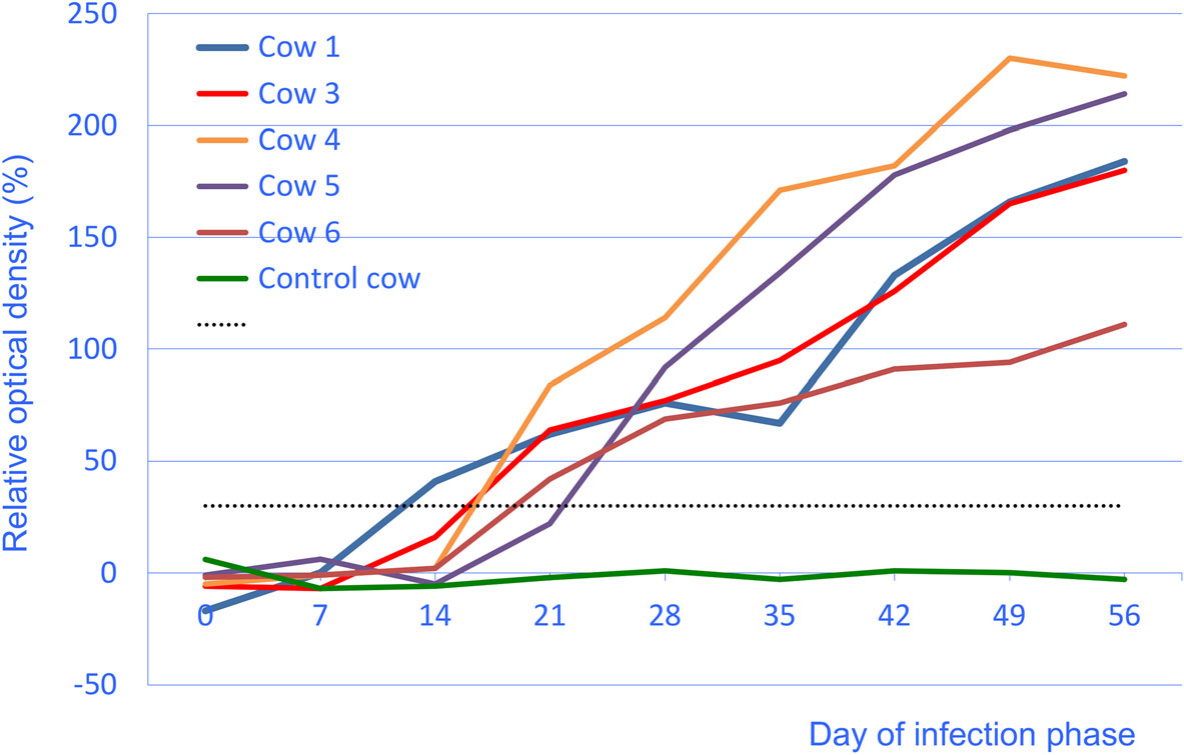
Relative optical density (OD) in the ELISA for pestivirus antibody in the serum of 5 cows inseminated with BD virus-infected semen and a control cow from day 0 to day 56 of the infection phase expressed as a percentage to the OD of a standard serum. Relative OD values > 30% (dotted line) are defined as positive

All cows were seronegative before insemination and during the first week of the infection phase. Seroconversion with OD values > 30% occurred in all experimental cows two to 4 weeks after insemination (first positive OD values between 41 and 92%; Fig. 5). The OD values further increased to between 111 and 222% by the time of slaughter. The OD of the control cow remained negative between − 7 and + 1%. The SNT was positive for BDV in all experimental cows and negative in the control cow (Table 1). Titres of specific neutralising BDV antibodies varied from 190 to 538. The SNT was negative (cows 3, 5, 6, and control) or very low (cows 1 and 4) for BVDV. The quotient of BDV and BVDV antibody titres ranged from 14 to > 32 in all experimental cows, which were clearly classified as infected with BDV. Neutralising antibodies against BDV and BVDV were not detected in the control cow.

**Table 1:** Relative OD values in the Ab-ELISA, SNT titres and quotients of BDV and BVDV SNT titres on day 56 of the infection phase in 5 cows inseminated with BDV-infected semen and in 1 control cow

Cow	OD value (%)	SNT BD virus	SNT BVD virus	Quotient of BD and BVD virus SNT titres
1	184	190	14	14
3	180	226	Negative^a^	≥ 28
4	222	538	17	32
5	214	226	Negative^a^	≥ 28
6	111	190	Negative^a^	≥ 24
Control	-3	Negative^a^	Negative^a^	NA

*NA* Not applicable

^a^ Limit of detection SNT ≤ 8

### Examination of uteri placentae, ovaries and fetuses

Four experimental cows (nos. 1, 4, 5, 6) were pregnant and one experimental and the control cow were not. All uteri, placentae, ovaries and fetuses were macroscopically and histologically normal, and the fetal organs and placentae did not yield pestiviral RT-PCR products.

### Pedigree analysis

The Eringer bull, the source of the pestivirus-free semen, was identified as the sire of all fetuses.

## Discussion

This study confirmed that cows inseminated with semen infected with BDV do not have overt clinical signs of illness [11] even with stringent health monitoring that included twice-hourly intraruminal temperature measurements and continuous recording of eating and rumination activities. The latter are sensitive criteria for the assessment of bovine wellbeing because sick cows usually have reduced rumination times, fewer regurgitated cuds and fewer chewing cycles per cud [22].

The most evident change in the leukogram was a significant decrease in the total leukocyte count on day 6 caused by lymphopenia, which was accompanied by normal neutrophil, eosinophil, basophil and monocyte numbers. Lymphopenia may be a response to stress-induced endogenous corticosteroid secretion [23] but can also occur in the acute phase of infection with viruses, *Ehrlichia*, mycoplasma and other microorganisms or with septicaemia [23]. Lambs [24] and pregnant ewes [25] experimentally infected with BDV had significant leukopenia from day 2 to days 6 and 5 post-infection, respectively, with a nadir on day 4 [25]. Differential leukocyte counts were not reported in those studies, but it can be assumed that the leukopenia was attributable to lymphopenia. Leukopenia and lymphopenia were also seen in calves experimentally infected with BVD virus strains of different virulence [13].

Except for one equivocal result, virus was not detected in the present study. Similarly, viraemia was not detected in calves [4, 9] and heifers in early pregnancy [6] housed with sheep persistently infected with BDV. A possible explanation for this is that transient pestivirus infections are characterised by short-lived and low-level viraemia, which makes detection of viral RNA almost impossible [4]. We believe that the weakly positive Ct value was due to minimal contamination in the laboratory because at the time of detection, the cow had already seroconverted. Seroconversion in cattle infected by sheep [4, 6] or other cattle persistently infected with BDV [9–11] has been reported. Eight heifers in early pregnancy co-housed with nine sheep persistently infected with BDV seroconverted 23 to 28 days after the start of exposure [6], and of nine calves co-housed with two persistently-infected sheep, six seroconverted after 36 to 72 days [10]. Six cows kept with a persistently-infected bull seroconverted after 20 to 40 days [10]. All of five cows had seroconverted by day 28 after insemination with BDV-infected semen [11], and the same observation was made in the present study.

In a previous study, three heifers in early pregnancy that were in contact with a calf persistently infected with BDV had persistently-infected fetuses and viral RNA in blood samples [10]. Five of eight heifers co-housed in early pregnancy with persistently-infected sheep aborted infected fetuses and three gave birth to healthy calves [6]. In another experiment, insemination of fertile heifers with BDV-infected semen did not result in pregnancies because of poor semen quality [11]. This problem was circumvented in the present study by using infected semen and virus-free semen, which resulted in pregnancies but, surprisingly, not in infected fetuses. This was in contrast to the results of a study in which pregnant heifers housed with a persistently-infected calf gave birth to persistently-infected offspring [10]. However, of 61 calves born to a BVDV-infected sire, only two (3.3%) were persistently infected [26]. Assuming a similar infection rate for BDV-infected semen, at least 30 cows would have had to be inseminated to generate one persistently-infected calf. Furthermore, acute BVDV infection only generates persistently-infected calves when it occurs from approximately day 30 to day 120 of pregnancy [27], whereas infection in the first month of pregnancy usually results in loss of pregnancy followed by return to estrus, or in a normal non-infected calf. Some researchers have speculated that the zona pellucida protects the conceptus from virus infection [28] or interferon-tau secreted by the trophoblast in the first 2 to 3 weeks of pregnancy has anti-viral properties [29]. Other factors including selection of a virus variant with different receptor requirements may also play a role in fetal infection [30]. To summarise, the findings of the present as well as earlier studies suggest that close contact with cattle [10] or sheep [4, 6, 20, 31] persistently infected with BDV plays a much bigger role in the pathogenesis of persistent infection than insemination with infected semen.

## Conclusions

The findings of the present study showed that artifical insemination using BDV-infected semen led to infection and seroconversion in all cows, but did not result in persistently-infected offspring. This mode of pestivirus infection therefore appears to be unlikely albeit not impossible in a virus-free herd, in contrast to infection through close contact with cattle or sheep persistently infected with BDV.

## Abbreviations

ABI: Applied biosystems
AEC: Amino-ethyl-carbazole
BDV: Border disease virus
BVD: Bovine viral diarrhoea
BVDV: Bovine viral diarrhea virus
Ct: Cycle threshold
EDTA: Ethylenediamintetraacetic acid
ELISA: Enzyme-linked immunosorbent assay
GAPDH: Glycerinaldehyde-3-phosphate-dehydrogenase
OD: Optical density
RNA: Ribonucleid acid
RT-PCR: Real-time polymerase chain reaction
SNT: Serum neutralization test
TCID: Tissue culture infective dose

## References

[1] Carlsson U, Belák K (1994). Border disease virus transmitted to sheep and cattle by a persistently infected ewe: epidemiology and control. Acta Vet Scand.

[2] Becher P, Orlich M, Shannon AD, Horner G, König M, Thiel HJ (1997). Phylogenetic analysis of pestiviruses from domestic and wild ruminants. J Gen Virol.

[3] Cranwell MP, Otter A, Errington J, Hogg RA, Wakeley P, Sandvik T (2007). Detection of border disease virus in cattle. Vet Rec..

[4] Krametter-Frötscher R, Benetka V, Möstl K, Baumgartner W (2008). Transmission of border disease virus from sheep to calves – a possible risk factor for the Austrian BVD eradication programme in cattle?. Wien Tierärztl Mschr.

[5] Hornberg A, Fernández SR, Vogl C, Vilček S, Matt M, Fink M, Köfer J, Schöpf K (2009). Genetic diversity of pestivirus isolates in cattle from western Austria. Vet Microbiol.

[6] Krametter-Frötscher R, Mason N, Roetzel J, Benetka V, Bago Z, Moestl K, Baumgartner W (2010). Effects of border disease virus (genotype 3) naturally transmitted by persistently infected sheep to pregnant heifers and their progeny. Vet Med.

[7] Strong R, La Rocca SA, Ibata G, Sandvik T (2010). Antigenic and genetic characterisation of border disease viruses isolated from UK cattle. Vet Microbiol.

[8] McFadden AMJ, Tisdall DJ, Hill FI, Otterson P, Pulford D, Peake J, Finnegan CJ, La Rocca SA, Kok-Mun T, Weir AM (2012). The first case of a bull persistently infected with border disease virus in New Zealand. N Z Vet J.

[9] Braun U, Reichle SF, Reichert C, Hässig M, Stalder HP, Bachofen C, Peterhans E (2014). Sheep persistently infected with border disease readily transmit virus to calves seronegative to BVD virus. Vet Microbiol.

[10] Braun U, Hilbe M, Janett F, Hässig M, Zanoni R, Frei S, Schweizer M (2015). Transmission of border disease virus from a persistently infected calf to seronegative heifers in early pregnancy. BMC Vet Res.

[11] Braun U, Frei S, Schweizer M, Zanoni R, Janett F (2015). Transmission of border disease virus to seronegative cows inseminated with infected semen. Res Vet Sci.

[12] Falkenberg SM, Ridpath J, Vander Ley B, Bauermann FV, Sanchez NCB, Carroll JA (2014). Comparison of temperature fluctuations at multiple anatomical locations in cattle during exposure to bovine viral diarrhea virus. Livest Sci.

[13] Chase CCL, Thakur N, Darweesh MF, Morarie-Kane SE, Rajput MK (2015). Immune response to bovine viral diarrhea virus – looking at newly defined targets. Anim Health Res Rev.

[14] Braun U, Trösch L, Nydegger F, Hässig M (2013). Evaluation of eating and rumination behaviour in cows using a noseband pressure sensor. BMC Vet Res.

[15] Braun U, Zürcher S, Hässig M (2015). Evaluation of eating and rumination behaviour in 300 cows of three different breeds using a noseband pressure sensor. BMC Vet Res.

[16] Stalder HP, Marti S, Flückiger F, Renevey N, Hofmann MA, Schweizer M (2017). Complete genome sequences of three border disease virus strains of the same subgenotype, BDSwiss, isolated from sheep, cattle, and pigs in Switzerland. Genome Announc.

[17] Schweizer M, Mätzener P, Pfaffen G, Stalder HP, Peterhans E (2006). “self” and “nonself” manipulation of interferon defense during persistent infection: bovine viral diarrhea virus resists alpha/beta interferon without blocking antiviral activity against unrelated viruses replicating in its host cells. J Virol.

[18] Canal CW, Strasser M, Hertig C, Masuda A, Peterhans E (1998). Detection of antibodies to bovine viral diarrhoea virus (BVDV) and characterization of genomes of BVDV from Brazil. Vet Microbiol.

[19] Bachofen C, Vogt HR, Stalder H, Mathys T, Zanoni R, Hilbe M, Schweizer M, Peterhans E (2013). Persistent infections after natural transmission of bovine viral diarrhoea virus from cattle to goats and among goats. Vet Res.

[20] Kaiser V, Nebel L, Schüpbach-Regula G, Zanoni RG, Schweizer M (2017). Influence of border disease virus (BDV) on serological surveillance within the bovine virus diarrhea (BVD) eradication program in Switzerland. BMC Vet Res.

[21] Hilbe M, Arquint A, Schaller P, Zlinszky K, Braun U, Peterhans E, Ehrensperger F (2007). Immunohistochemical diagnosis of persistent infection with bovine viral diarrhea virus (BVDV) on skin biopsies. Schweiz Arch Tierheilk..

[22] Braun U, Tschoner T, Hässig M, Nuss K (2017). Eating and rumination behaviour in cows with traumatic reticuloperitonitis. Schweiz Arch Tierheilk..

[23] Tornquist S, Rigas J, Weiss DJ, Wardrop KJ (2010). Interpretation of ruminant leukocyte responses. Schalm’s Veterinary Hematology.

[24] Thabti F, Fronzaroli L, Dlissi E, Guibert JM, Hammami S, Pepin M, Russo P (2002). Experimental model of border disease virus infection in lambs: comparative pathogenicity of pestiviruses isolated in France and Tunisia. Vet Res.

[25] García-Pérez AL, Minguijón E, Estévez L, Barandika JF, Aduriz G, Juste RA, Hurtado A (2009). Clinical and laboratorial findings in pregnant ewes and their progeny infected with border disease virus (BDV-4 genotype). Res Vet Sci.

[26] Kirkland PD, Mackintosh SG, Moyle A (1994). The outcome of widespread use of semen from a bull persistently infected with pestivirus. Vet Rec.

[27] Brownlie J (1990). The pathogenesis of bovine virus diarrhoea virus infections. Rev Sci Tech Off Int Epiz.

[28] Brock KV, Grooms DL, Givens MD, Goyal SM, Ridpath JF (2005). Reproductive disease and persistent infections. Bovine viral diarrhea virus: diagnosis, management and control.

[29] Schweizer M, Peterhans E (2014). Pestiviruses. Annu Rev Anim Biosci.

[30] Swasdipan S, McGowan M, Phillips N, Bielefeldt-Ohmann H (2002). Pathogenesis of transplacental virus infection: pestivirus replication in the placenta and fetus following respiratory infection. Microb Pathog.

[31] Braun U, Bachofen C, Schenk B, Hässig M, Peterhans E (2013). Investigation of border disease and bovine virus diarrhoea in sheep from 76 mixed cattle and sheep farms in eastern Switzerland. Schweiz Arch Tierheilk.

[32] Stöber M, Dirksen G, Gründer HD, Stöber M (1990). Körpertemperatur. Die klinische Untersuchung des Rindes.

[33] Stöber M, Gründer HD, Dirksen G, Gründer HD, Stöber M (1990). Weisses Blutbild. Die klinische Untersuchung des Rindes.

