# Correction to: Insemination with border disease virus-infected semen results in seroconversion in cows but not persistent infection in fetuses

**DOI:** 10.1186/s12917-018-1497-x

**Published:** 2018-06-11

**Authors:** Ueli Braun, Fredi Janett, Sarah Züblin, Michèle von Büren, Monika Hilbe, Reto Zanoni, Matthias Schweizer

**Affiliations:** 1 Department of Farm Animals, Vetsuisse Faculty, University of Zurich, Zurich, Switzerland; 2 Department of Farm Animals, Vetsuisse Faculty, University of Zurich, Zurich, Switzerland; 3 Institute of Veterinary Pathology, Vetsuisse Faculty, University of Zurich, Zurich, Switzerland; 4 Institute for Virology and Immunology, and Department of Diseases and Pathobiology, Vetsuisse Faculty, University of Bern, Bern, Switzerland

## Correction

The original article [1] contained an error whereby a co-author, Sarah Züblin had their name displayed incorrectly. This error has now been corrected.
